# Physiological and sedative responses in *Phrynops geoffroanus* (Schweigger, 1812): Comparison of two anesthetic protocols using dexmedetomidine and ketamine

**DOI:** 10.1007/s11259-026-11461-4

**Published:** 2026-08-21

**Authors:** Ademar Francisco Fagundes Meznerovvicz, Ana Letícia Rodrigues Marques, Marina Marangoni, Ana Paula Gomes Lustosa, Rafael Martins Valadão, Paulo Henrique Braz

**Affiliations:** 1 Federal University of Fronteira Sul, Edmundo Gaievski Avenue, 1000, Highway BR 182 - Km 466, PO Box 253, Rural Area, Realeza, Paraná 85770-000 Brazil; 2 Chico Mendes Institute for Biodiversity Conservation (ICMBio), National Center for Research and Conservation of Reptiles and Amphibians, Goiás, Goiânia Brazil

**Keywords:** Chemical restraint, Dissociative anesthesia, Chelonians, Sedation

## Abstract

This study evaluated the anesthetic combination of dexmedetomidine and ketamine in free-ranging *Phrynops geoffroanus*, assessing cardiopulmonary and sedative variables, as well as pharmacological reversal with atipamezole. Twenty-five adult individuals were allocated into two groups: HG (dexmedetomidine 0.1 mg/kg + ketamine 10 mg/kg; *n* = 13) and LG (dexmedetomidine 0.05 mg/kg + ketamine 10 mg/kg; *n* = 12). Heart rate, respiratory rate, and cloacal temperature, along with motor and behavioral reflexes and venous blood gas analysis, were evaluated at baseline, during chemical restraint, and throughout recovery. No significant differences were observed between groups at the same time points for heart rate, respiratory rate, or cloacal temperature, indicating physiological stability at both dosages. In the HG group, respiratory rate progressively decreased from baseline (39 ± 13 [12–64]) to 7 ± 12 [0–42] between 35 and 45 min after drug administration, with recovery to 36 ± 10 [17–56] fifteen minutes following atipamezole administration. Reflex assessment demonstrated deeper sedation in the HG group, with significant differences in head tone, limb tone, righting reflex, and palpebral reflex, whereas cloacal reflex and jaw tone did not differ between doses. Pharmacological reversal restored physiological parameters to near-baseline values within 25 min. Venous blood gas analysis revealed mild and transient changes in pH and pCO₂ in both groups, without clinically relevant consequences. In conclusion, dexmedetomidine at doses of 0.1 mg/kg and 0.05 mg/kg, combined with ketamine (10 mg/kg) and administered at an ambient temperature of 28 °C, is safe for *P. geoffroanus*. The higher dose was associated with deeper sedation, making it suitable for field procedures and clinical handling.

## Introduction

Anesthesia in chelonians presents specific challenges related to the physiological and anatomical particularities of this group, including the absence of a diaphragm and intercostal muscles, the restriction of the coelomic cavity by the carapace, and the ability to maintain prolonged apnea, factors that complicate anesthetic management (Bennett 1998; Heard 2001; O’Malley 2018; Olsson and Simpson 2018; Perpiñán 2018).

In this context, pharmacological protocols that provide adequate chemical restraint with minimal cardiorespiratory compromise are essential. Dexmedetomidine, a highly selective alpha-2 adrenergic agonist, promotes sedation and analgesia by reducing neuronal excitability, while ketamine, an N-methyl-D-aspartate (NMDA) receptor antagonist, is widely used in reptiles due to its efficacy and relatively limited cardiorespiratory depression (Longley 2008; Giovannitti et al. 2015; Rankin 2017; Bao and Tang 2020; Sayers and Kubiak 2020).

The combination of these drugs aims to potentiate sedative effects and improve the quality of anesthetic recovery, and has been evaluated in chelonian species via different routes of administration, including intramuscular, intranasal, and intracloacal routes (Schnellbacher et al. 2012; Cermakova et al. 2017; Morici et al. 2017). To reverse the effects induced by dexmedetomidine, atipamezole is commonly used in doses higher than those of the alpha-2 agonist, promoting a faster and more predictable recovery; however, considerable variability persists among anesthetic protocols for chelonians (Mosley 2005; Schnellbacher et al. 2012; Cermakova et al. 2017).

*Phrynops geoffroanus* (Schweigger, 1812), commonly known as the Geoffroy’s side-necked turtle, belongs to the order Testudines and the family Chelidae. This species has a wide distribution in South America and occurs throughout most of Brazil, except for the Pampa biome (TTWG 2025; Brito et al. 2025). It is a semi-aquatic, diurnal species that generally exhibits docile behavior and a high capacity to adapt to environments with low oxygen availability and urbanized areas, being considered an important environmental bioindicator (Souza and Abe 2000; Pulcherio et al. 2022; Brito et al. 2025).

In *P. geoffroanus*, anesthetic studies are scarce and largely focused on benzodiazepines, intravenous anesthetics, or dissociative agents administered through different routes (Santos et al. 2009, 2012a, b; Bragagnoli et al. 2020). Reports of route-related complications, such as accidental submeningeal propofol injection, highlight the need for safer alternatives (Quesada et al. 2010). Given the heterogeneity of existing protocols, the intramuscular route stands out as a practical option under field conditions, especially in high-density settings like Wildlife Screening Centers (CETAS), due to its ease of administration and rapid onset (Santos et al. 2025).

Thus, the present study aimed to evaluate the association of dexmedetomidine and ketamine, analyzing cardiopulmonary and sedative variables, as well as the reversal of dexmedetomidine with atipamezole, in wild *Phrynops geoffroanus*.

## Materials and methods

### Animals, capture and post-procedural

This is a prospective, comparative, experimental study employing simple random allocation to assign individuals to test groups. It was conducted with 25 free-ranging adults *Phrynops geoffroanus*, comprising both males and females, depending on capture feasibility within Iguaçu National Park, comparing two different dexmedetomidine dosages (0.1 mg/kg in the HG group and 0.05 mg/kg in the LG group) combined with a fixed dose of ketamine (10 mg/kg). Trap locations were selected based on field inspections and prior records of the species occurrence.

Animals were captured manually and using funnel traps (“covo” type), measuring 100 cm in length and 50 cm in height, with 2 cm mesh size and a 25 cm funnel opening. Traps were simultaneously installed along the margins of pre-established aquatic environments during the morning (08:00 h) and inspected and removed the following morning (08:00 h), remaining active for a total of 24 h.

To prevent drowning, traps were installed with approximately 30% of their structure emergent, allowing respiration of captured individuals. The distance between traps was approximately 50 m. All non-target organisms (not included in the collection permit) were immediately released at the capture site.

Inspections were performed in the morning, when turtles were removed from traps, identified, labeled, and placed in individual cloth bags. Subsequently, they were transported to the ICMBio research base, where temporary handling was conducted in accordance with ICMBio Ordinance No. 748/2022. Animals were individually housed in light-colored, fine cotton bags, slightly moistened, ensuring adequate ventilation and comfort.

All procedures involving restraint, housing, transport, post-procedural care, and release followed the recommendations of the *Brazilian Guide for the Production*,* Maintenance*,* or Use of Animals in Teaching or Scientific Research Activities* of the National Council for the Control of Animal Experimentation (CONCEA) (Lustosa et al. 2023). After completion of procedures, animals were released back into the wild at the original capture sites.

### Anesthesia and monitoring

Prior to inclusion in the study, the animals underwent a physical examination, including assessment of hydration status and body condition score, as well as the collection of biological samples for baseline blood gas analysis. Animals exhibiting dehydration, poor body condition, visible lesions, or any condition contraindicating chemical restraint were excluded from the study; no adult animals met the exclusion criteria. Juvenile animals whose sex could not be determined were also excluded.

Of the 25 animals, 16 were females and nine were males. The animals were assigned to two experimental groups: a high-dose dexmedetomidine group (HG) (*n* = 13; eight females and five males) and a low-dose dexmedetomidine group (LG) (*n* = 12; eight females and four males). Body weight differed between the sexes: females weighed 582 ± 291 (310–1280) g, and males weighed 1,062 ± 814 (250–2310) g. Dexmedetomidine (Dexdomitor^®^, Orion Pharma, Espoo, Finland) was administered in combination with ketamine (Cetamin^®^, Syntec, Brazil), followed by reversal with atipamezole (Antisedan^®^, Orion Pharma, Espoo, Finland) as follows:

- Group 1 (HG): dexmedetomidine (0.1 mg/kg) combined with ketamine (10 mg/kg), reversal with atipamezole (0.1 mg/kg).
- Group 2 (LG): dexmedetomidine (0.05 mg/kg) combined with ketamine (10 mg/kg), reversal with atipamezole (0.05 mg/kg).

The drugs were administered immediately after the baseline physiological assessment, via intramuscular injection using a sterile hypodermic needle (0.45 × 13 mm) into the left biceps brachii muscle, following appropriate cleaning and antisepsis of the injection site.

The animals from both groups were evaluated at five-minute intervals to determine heart rate (HR), respiratory rate (RR), and cloacal temperature (CT), as well as spontaneous movements (SM) was performed for observacion visual, head righting reflex (HRR), head tone (HT), limb tone (LT) jaw tone (JT) was performed through tactile manipulation and extension of the head, limbs and attempted mouth opening for JT; palpebral reflex (PR) was gentle eye contact, for the cloacal reflex (CR), was assessed simultaneously, with the using a thermometer.

Anesthetic monitoring included the use of a multiparametric monitor (RMZ1200vet, RZVet, São Paulo, Brazil) for CT, a veterinary ultrasonic Doppler device (Medmega DV-610 V, São Paulo, Brazil) for HR placement at the thoracic inlet, and RR assessment by visual observation of thoracic limb, neck, or pelvic limb pumping movements. Ambient temperature was recorded using a thermo-hygrometer (HTC-2).

The observed reflexes (SM, CR, HRR, PR, HT, LT, and JT) were classified according to a three-point scale: 1 – present;2 – reduced; 3 – absent (Santos et al. 2009; Schnellbacher et al. 2012; Bragagnoli et al. 2020; Heniff et al. 2023).

Chemical restraint lasted 45 min following the initial administration. At the end of this period (M45), after parameter assessment, reversal was performed via intramuscular injection at the same site using atipamezole (Divers et al. 2022), with monitoring continuing up to 70 min post-administration to track the return of consciousness. The same parameters were monitored during recovery (from M45 to M70). No animal exhibited adverse events, such as post-anesthetic excitement.

### Venous Blood Gas Analysis

Five animals from each group (HG, *n* = 5; LG, *n* = 5), were selected for pre- and post-anesthetic venous blood gas analysis. Blood samples (approximately 0,5 mL) were collected via dorsal occipital venous sinus puncture using a disposable 3 mL heparinized syringe and a 30 × 7 needle.

Blood gas analysis was performed, on average, 3 h prior to the administration of the chemical restraint protocol (pre-anesthetic; P0) and after anesthetic recovery, approximately 50 min following reversal (post-anesthetic; P1), to detect potential alterations induced by the chemical restraint protocol.

Analyses were performed using a portable EPOC^®^ Blood Gas Analyzer (Siemens Healthineers), employing three measurement methods depending on the parameter assessed: potentiometry (Na⁺, K⁺, Ca²⁺, pH, and pCO₂) and amperometry (Glu, Lac, and pO₂). Each analysis was conducted individually using a test card containing calibrators, controls, and electrodes.

Measured and calculated blood gas parameters included base excess in blood (BE [b]), base excess in extracellular fluid (BE [ecf]), calcium (Ca), chloride (Cl), bicarbonate concentration (cHCO₃⁻), oxygen saturation (Cso₂), glucose (Glu), potassium (K), lactate (Lac), sodium (Na), partial pressure of carbon dioxide (pCO₂), hydrogen potential (pH), partial pressure of oxygen (pO₂), and total carbon dioxide (tCO₂).

Corrections for pH, pCO₂, and pO₂ were applied based on mean ambient temperature (TA), with P0 at 25 °C and P1 at 28 °C. The variables cHCO₃⁻ and tCO₂ were recalculated using temperature-based formulas described by Stabenau and Heming (1993), Anderson et al. (2011), and Adamovicz et al. (2018):

$$\mathrm{pH}\left(\mathrm{T_A}\right)=\mathrm{pH_I}-0{,}0147\times\left(\mathrm{T_A}-37\right)+0{,}0065\times\left(7{,}4-\mathrm{pH_I}\right)\times\left(\mathrm{T_A}-37\right)$$

$${\mathrm{pO}}_2\left(\mathrm{TA}\right)={\mathrm{pO}}_2\mathrm I\times10^{\left(-0,0058\times\left(\mathrm{TA}-37\right)\right)}$$

$${\mathrm{pCO}}_2\left(\mathrm{TA}\right)={\mathrm{pCO}}_2\mathrm I\times10^{\left(0,0019\times\left(\mathrm{TA}-37\right)\right)}$$

$$\alpha CO_2=\left(9{,}174\times10^{-2}\right)-\left(3{,}269\times10^{-3}\times TA\right)+\left(6{,}364\times10^{-5}\times TA^2\right)-\left(5{,}378\times10^{-7}\times TA^3\right)$$

$$pK_a=6{,}398-\left(1{,}341\times10^{-2}\times TA\right)+\left(2{,}282\times10^{-4}\times TA^2\right)-\left(1{,}516\times10^{-6}\times TA^3\right)-\log\left(1{,}011+10^{pH(TA)+0{,}011\times TA-10{,}241}+10^{pH(TA)+0{,}001\times TA-8{,}889}\right)$$

$${\mathrm{HCO}}_3^-\left(\mathrm{TA}\right)=\alpha{\mathrm{CO}}_2\times{\mathrm{pCO}}_2\left(\mathrm{TA}\right)\times10^{\left(\mathrm{pH}\left(\mathrm{TA}\right)-{\mathrm{pK}}_{\mathrm a}\right)}$$

$${\mathrm{tCO}}_2\left(\mathrm{TA}\right)={\mathrm{HCO}}_3^-\left(\mathrm{TA}\right)+\left(\alpha{\mathrm{CO}}_2\times{\mathrm{pCO}}_2\left(\mathrm{TA}\right)\right)$$

#### Statistical Analysis

Descriptive statistics (mean ± SD, minimum, and maximum values) were used to summarize the data. Heart rate (HR), respiratory rate (RR), and cloacal temperature (CT) were recorded every five minutes, and data were consolidated into15-minute intervals and 10 min for the last measurement interval. Post-baseline time points represent data compiled at 15-min intervals (M5–15 to M50–60) and at 10-min intervals for M65–70, as defined in the statistical analysis.

Based on normality assessment using the Shapiro–Wilk test, repeated-measures ANOVA was applied to evaluate differences in HR, RR, and CT. Homogeneity of variances was assessed using Levene’s test. When violations of sphericity were detected, the Greenhouse–Geisser correction was applied.

Reflex variables were analyzed using their complete datasets, without temporal aggregation. An independent-samples t-test was used to compare reflex responses between the HG and LG groups, with results graphically represented to visualize the timing of reflex reduction, absence, and recovery throughout the monitoring period.

For venous blood gas analysis, normality was assessed using the Shapiro–Wilk test. Paired-samples t-tests were applied to evaluate differences between time points within the same group, and independent-samples t-tests were used to compare differences between groups at the same time points.

All statistical analyses were performed using JASP software (version 0.19.3.0) (JASP 2025). Statistical significance was set at *p* < 0.05.

## Results

### Physiological Parameters

Heart rate (HR), respiratory rate (RR), and total cloacal temperature (CT) did not differ significantly between groups at the same time points after correction for sphericity and homogeneity of variances. Descriptive data for these physiological parameters and the significant results (*p* < 0.05) of the post hoc repeated-measures ANOVA, referring to comparisons among time points within each experimental group (without between-group comparisons), are presented in Table 1. The time points described represent the mean ± SD [min.–max.].

**Table 1:** Physiological parameters: heart rate

Groups		Baseline	M5-15	M20-30	M35-45	M50-60	M65-70
HR	HG	50 ± 15 [20–76] ^abc^	46 ± 6 [28–53] ^a^	40 ± 5 [26–48] ^b^	38 ± 5 [30–49] ^b^	50 ± 10 [28–64] ^a^	56 ± 13[24–72] ^c^
	LG	56 ± 5 [46–64] ^a^	45 ± 4 [40–53] ^b^	38 ± 2 [34–44] ^c^	34 ± 4 [25–41] ^d^	49 ± 10 [31–65] ^ab^	56 ± 12 [34–70] ^a^
RR	HG	39 ± 13 [12–64] ^a^	6 ± 7 [0–26] ^b^	7 ± 14 [0–48] ^b^	7 ± 12 [0–42] ^b^	36 ± 10 [17–56] ^a^	42 ± 14 [25–61] ^a^
	LG	35 ± 18 [0–64] ^a^	7 ± 8 [0–20] ^b^	7 ± 12 [0–44] ^b^	8 ± 9 [0–31] ^b^	29 ± 10 [12–42] ^a^	34 ± 12 [12–52] ^a^
CT	HG	26 ± 1 [24–28] ^a^	26 ± 1 [24–28] ^a^	27 ± 1 [25–28] ^b^	27 ± 0,8 [25–28] ^b^	27 ± 0,8 [25–28] ^b^	27 ± 0,8 [25–28] ^b^
	LG	26 ± 1 [24–28]	27 ± 0,9 [25–29]	27 ± 1 [25–29]	27 ± 0,9 [25–29]	27 ± 0,8 [25–29]	27 ± 0,8 [26–29]

(HR; bpm; mean ± SD [min–max]), respiratory rate (RR; breaths/min; mean ± SD [min–max]), and cloacal temperature (CT; °C; mean ± SD [min–max]) of *Phrynops geoffroanus* subjected to chemical restraint with dexmedetomidine and ketamine. Pharmacological reversal of dexmedetomidine was performed after M35-45 min. Baseline values were recorded before drug administration. Post-baseline time points represent data compiled at 15-min intervals (M5–15 to M50–60) and at 10-min intervals for M65–70, as defined in the statistical analysis. HG, high-dose dexmedetomidine group; LG, low-dose dexmedetomidine group. Different lowercase letters indicate significant differences between time points (*p* < 0.05)

### Reflex assessment

The HG and LG groups showed statistically significant differences at specific time points. For the head tone (HT) parameter, differences were observed at M20 (*p* = 0.010), M25 (*p* < 0.001), M35 (*p* < 0.001), M40 (*p* < 0.001), and M45 (*p* = 0.008). Spontaneous movement (SM) differed between groups at M35 (*p* = 0.025). The head righting reflex (HRR) showed significant differences at M35 (*p* = 0.008), M40 (*p* < 0.001), and M45 (*p* = 0.018), whereas the palpebral reflex (PR) differed only at M45 (*p* = 0.024).

**Fig. 1:**
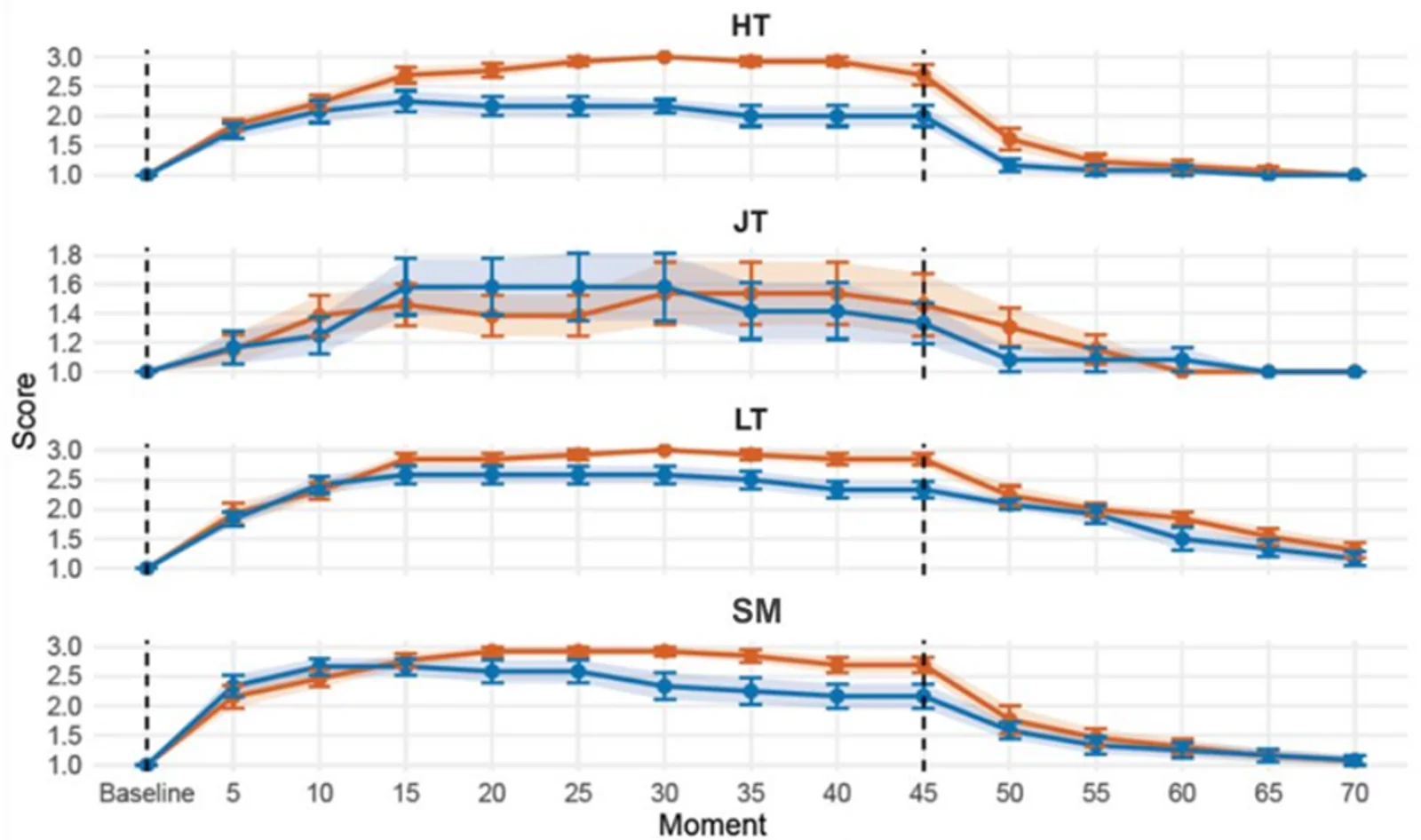
Behavioral parameters and reflexes observed during anesthetic monitoring of *Phrynops geoffroanus* subjected to the anesthetic protocol. Pharmacological reversal of dexmedetomidine was performed at 45 min (dashed line). The orange line represents the high-dose group (HG), and the blue line represents the low-dose group (LG). Scores were recorded over time to assess anesthetic depth and recovery. The baseline time point corresponds to the moment immediately prior to drug administration. From this point, variables were recorded at regular five-minute intervals. SM, spontaneous movements; HT, head tone; LT, limb tone; JT, jaw tone

**Fig. 2:**
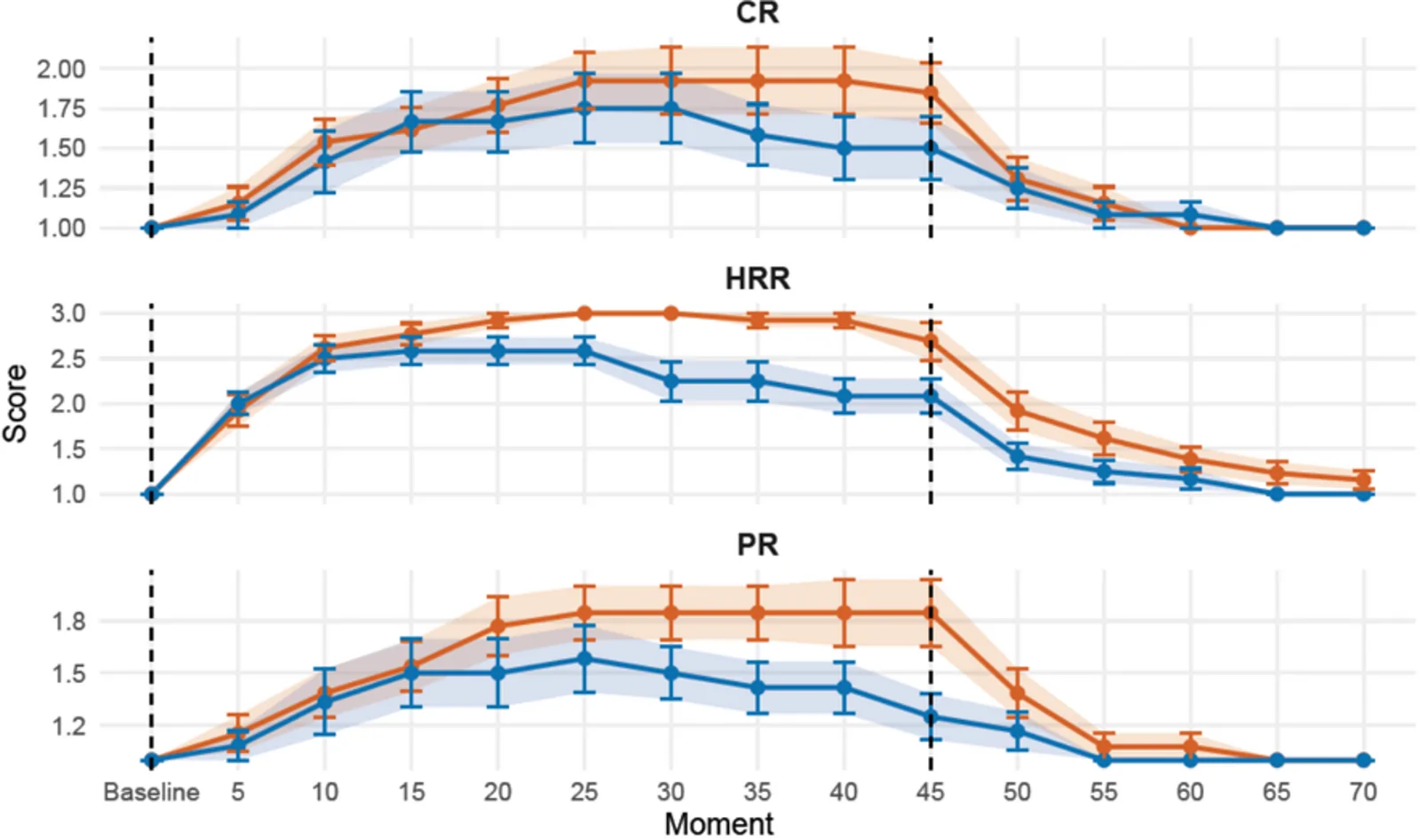
Behavioral parameters and reflexes during anesthetic monitoring of *Phrynops geoffroanus*. Pharmacological reversal of dexmedetomidine was performed at 45 min (dashed line). The orange line represents the high-dose group (HG) and the blue line the low-dose group (LG). The baseline time point corresponds to the moment immediately prior to drug administration. From this point, variables were recorded at regular five-minute intervals. CR, cloacal reflex; HRR, head righting reflex; PR, palpebral reflex

Regarding limb tone (LT), significant dose-related differences were observed at M35 (*p* = 0.023), M40 (*p* = 0.011), and M45 (*p* = 0.011). The cloacal reflex (CR) and jaw tone (JT) did not show significant differences between the evaluated doses. These results are graphically presented in Figs. 1 and 2.

### Blood gas parameters

Values were obtained from blood samples collected from *n* = 5 animals per group. Regarding within-group temporal differences, the HG group showed significant differences between P0 and P1 for pH (*p* = 0.040), pCO₂ (*p* = 0.015) and Cso_2_ (*p* = 0.049). In the LG group, significant temporal differences were observed for pCO₂ (*p* = 0.009) and chloride (Cl) (*p* = 0.049). Between-group comparisons at the same temporal point revealed significant differences at P0 between HG and LG for pCO₂ (*p* = 0.017) and calcium (Ca) (*p* = 0.039), however, no significant differences between groups were observed at P1. Descriptive values are presented in Table 2.

**Table 2:** Blood gas parameters of *Phrynops geoffroanus* at pre-anesthetic (P0) and post-anesthetic (P1) time points (HG, *n* = 5; LG, *n* = 5)

Values	Period	Groups	
		HG	LG
pH (TA)	P0	7.435 ± 0.071 ^a^	7.577 ± 0.119
	P1	7.676 ± 0.168 ^b^	7.704 ± 0.096
pCO_2_(TA)	P0	57.8 ± 10.393 ^A a^	41.420 ± 6.353 ^B a^
	P1	31.72 ± 11.355 ^b^	28.94 ± 6.222 ^b^
pO_2_(TA)	P0	67.96 ± 14	72.1 ± 13.539
	P1	70.52 ± 5.145	66.88 ± 8.819
cHCO_3_(TA)	P0	48.14 ± 6.398	48.46 ± 11.329
	P1	47.260 ± 9.906	43.76 ± 5.477
BE (ecf)	P0	2.9 ± 3.168	−1.52 ± 5.569
	P1	4.54 ± 3.609	3.12 ± 1.395
BE (b)	P0	2.8 ± 2.772	−1.36 ± 5.003
	P1	3.920 ± 3.194	2.580 ± 1.238
Cso_2_	P0	82.1 ± 9.867 ^a^	90.62 ± 9.075
	P1	94.12 ± 2.774 ^b^	93.820 ± 2.16
Na	P0	136.8 ± 2.28	133.8 ± 3.834
	P1	136.2 ± 4.147	134.8 ± 2.588
K	P0	3.560 ± 0.321	3.66 ± 0.619
	P1	3.2 ± 0.2	3.2 ± 0.515
Ca	P0	6.0 (6.0–6.2) ^A^	6.56 ± 0.445 ^B^
	P1	5.82 ± 0.37	6.16 ± 0.577
Cl	P0	94.8 ± 4.025	95.4 ± 3.286 ^a^
	P1	95.8 ± 4.266	98 ± 4.183 ^b^
tCO_2_(TA)	P0	50.520 ± 6.625	50.16 ± 11.206
	P1	48.460 ± 10.310	44.78 ± 5.694
Glu	P0	178.6 ± 70.918	142 (138–300)
	P1	203.8 ± 84.138	201.8 ± 75.629
Lac	P0	71.84 ± 24.605	100.04 ± 42.209
	P1	76.66 ± 18.215	72.980 ± 16.9

Data are presented as mean ± SD or median (min–max), according to distribution. BE (b), base excess in blood; BE (ecf), base excess in extracellular fluid; cHCO₃⁻, bicarbonate concentration; Cso₂, oxygen saturation; HG, high-dose group; Glu, glucose; Lac, lactate; LG, low-dose group; pCO₂, partial pressure of carbon dioxide; pO₂, partial pressure of oxygen; TA, Corrections applied based on mean ambient temperature; tCO₂, total carbon dioxide. Different uppercase letters indicate differences between groups, and different lowercase letters indicate differences between time points (*p* < 0.05)

## Discussion

The combination of dexmedetomidine and ketamine followed by reversal with atipamezole was effective in both groups, with the HG group indicated for procedures requiring deep sedation and the LG group for procedures requiring light to moderate sedation in *P. geoffroanus.* The same combination has been evaluated in several studies using the intranasal route (Schnellbacher et al. 2012; Cermakova et al. 2017) and the intracloacal route (Morici et al. 2017), with reports of its use at the doses applied in the present study (Divers et al. 2022).

To date, its use in *P. geoffroanus* and the response of this protocol to reversal with dexmedetomidine have not been reported. This gap is particularly relevant considering the wide geographic distribution of *P. geoffroanus*, its abundance throughout Brazil, and its high population density, factors that favor its inclusion in clinical, experimental, and conservation-related procedures. Thus, the results of this study provide new contributions to the anesthesiology of neotropical turtles, offering support for the development of safer and more predictable protocols applicable to the management of Brazilian wildlife (Brito et al. 2025).

Studies involving *P. geoffroanus* in Brazilian literature have predominantly employed drugs such as ketamine, etomidate, midazolam, and propofol, focusing on sedation parameters assessed using subjective scales that evaluate the presence or absence (1–2) or presence, reduction, and absence (1–3) of specific parameters (Santos et al. 2009, 2012a, b; Cordeiro 2019; Bragagnoli et al. 2020), as well as variations in physiological parameters, most frequently HR, RR, and CT, in addition to ambient temperature. For *P. geoffroanus*, ambient temperatures of 28 °C (Bragagnoli et al. 2020) have been reported for anesthesia. Conversely, a study conducted in Mato Grosso do Sul, Brazil, reported a preferred temperature range between 24.31 °C and 30.61 °C (Emidio and Barradas 2017).

The reduction of RR is commonly observed with the use of alpha-2 agonists in anesthetic protocols. This effect has been reported in Chelonoidis carbonaria using the same HG protocol doses combined with 1 mg/kg of midazolam (Eshar et al. 2021). A similar response was observed in Trachemys scripta scripta, consistent with the progressive decrease until reversal observed in the present study (Schnellbacher et al. 2012; Morici et al. 2017). The RR of *P. geoffroanus* under normoxic conditions is low compared to other species, such as *Podocnemis unifilis* (Cordeiro et al. 2016). There are no data available for comparison with other species of the same genus, suggesting that the association with an inherently low RR may predispose this species to bradypnea or apnea during sedation (Cordeiro et al. 2016).

HR showed a progressive reduction over time following application of the chemical restraint protocol, with a subsequent increase after reversal with atipamezole, returning to values similar to baseline. A similar pattern was described in *Trachemys scripta elegans* using 0.2 mg/kg of intranasal dexmedetomidine, a dose twice that used in the HG protocol, combined with the same ketamine dose (Cermakova et al. 2017). In that study, this HR variation pattern was observed in both experimental groups. In contrast, studies using 0.2 mg/kg of dexmedetomidine in *Trachemys scripta scripta* reported an increase or maintenance of baseline HR over time, possibly related to species-specific responses (Schnellbacher et al. 2012; Morici et al. 2017).

The HG group presented higher scores on the subjective sedation scale (1–3) compared to the LG group, a result also illustrated in Figs. 1 and 2. In both groups, mean scores of 2 in the LG group and 3 in the HG group were observed for head tone, palpebral reflex, and mandibular tone, indicating reduction or absence of these responses. These findings contrast with those reported by Morici et al. (2017), who, despite using dexmedetomidine doses twice as high as those in the HG protocol, described similar sedation levels, with a predominance of score 2 at 45 min, representing decreased or absent head and neck tone, present palpebral reflex, and present mandibular tone. In the present study, the HG protocol promoted greater suppression of the evaluated parameters despite the lower dose, suggesting a more pronounced sedative effect relative to the dose used.

The HG group exhibited profound reflex suppression, with a total absence of spontaneous movements between M20 and M35, the head-righting reflex (HRR) and limb tone (LT) reached a score of 3 (absence of reflexes) at time points such as M25–M30 and M30, whereas the LG group showed only a reduction (Score 2). Spontaneous movements and head-righting reflexes provide useful information in chelonians, and flaccidity of the head and limbs was categorized as moderate sedation, with no return to the retracted position following manipulation (Schnellbacher et al. 2012; Sladky and Mans 2012). These findings indicate moderate sedation in the LG group and profound sedation in the HG group. However, digital pinching of the limbs to assess deeper reflexes was not performed, only tactile manipulation of the limbs and head was evaluated.

The cloacal reflex was reduced (score 2), showing a more marked decrease in the HG group compared to the LG group, followed by complete recovery within 25 min of dexmedetomidine reversal with atipamezole. However, this parameter was not evaluated in other studies using the same chemical restraint protocol, nor in other anesthetic studies involving *P. geoffroanus*. This recovery pattern was consistent with that observed for most of the reflexes evaluated. Other studies reported a complete return to baseline levels 18.9 ± 7 min after reversal (Schnellbacher et al. 2012) using a dose ten times higher. Similar results were reported by Cermakova et al. (2017). Recovery time may be associated with species-specific responses and can vary.

The slower reflex recovery observed in this study, compared to reports by Cermakova et al. (2017), may be attributed to the atipamezole ratio used (1:1), which differs from the tenfold higher dose commonly recommended in the literature. Due to its nature as a competitive antagonist, atipamezole is frequently used at doses much higher than those of the alpha-2 agonist to ensure safe reversal and prevent resedation, doses ten times higher are widely reported in studies involving chelonians (Schnellbacher et al. 2012; Fleming et al. 2008; Harms et al. 2014; Cermakova et al. 2017; Masi et al. 2023). Thus, it is concluded that, although the current protocol is effective, increasing the atipamezole dose could reduce the total anesthetic recovery time for this species.

Hemogasometric values of pH (TA) for the HG group and pCO₂(TA) for both groups differed between P0 and P1 within the same group, showing higher pH (TA) and lower pCO₂(TA) values at the post-chemical restraint moment. There are no standardized reference values in the literature for this species to allow direct comparisons. However, the increase in pH (TA) at P1 compared to P0 in the HG group may be attributed to an increase in RR after reversal, leading to decreased pCO₂(TA) and stable bicarbonate levels at both time points, resulting in increased pH (DiBartola 2012).

The HG group exhibited higher pCO_2_(TA) values at time point P0 compared to the LG group (*p* = 0.017), but this difference disappeared at P1, indicating that both protocols result in a similar blood gas profile following recovery. The significant reduction in pCO_2_(TA) observed during the post-anesthetic period in both groups mirrors findings in *Chelonia mydas* (Justo et al. 2023), where a drop in pCO_2_ relative to baseline values was also recorded. However, while Justo et al. (2023) attributed this to mechanical ventilation-induced hyperventilation, the decrease in the present study may be associated with spontaneous ventilatory recovery triggered by atipamezole reversal, evidenced by the marked increase in respiratory rate (RR) during the M50–60 and M60–70 intervals (Table 1). In both scenarios, hyperventilation facilitated CO_2_ elimination, contributing to the rise in pH observed during the post-anesthetic period (DiBartola 2012).

Blood gas analysis values for *Rheodytes leukops* have been reported in the literature, with pH values corrected to a temperature of 25 °C showing higher values compared to the P0 values in *P. geoffroanus* in the present study (Gordos et al. 2004). Venous hemogasometric parameters in free-ranging *Terrapene carolina carolina* showed lower pH values in summer compared to other seasons, in contrast, P0 values in both groups of the present study were higher (Adamovicz et al. 2018). The pH values (7.519) from six individuals of *Phrynops hilarii* were established at 25 °C, along with pCO₂ values, which were similar to P0 values in the LG group of the present study, these animals were also maintained at an ambient temperature of 25 °C during the experiment (Reischl et al. 1984).

The lower pO_2_(TA) values observed in the LG group should be interpreted taking into account the nature of the sample. Unlike studies using arterial blood, the samples analyzed here were collected from the dorsal occipital venous sinus, venous blood reflects the balance between oxygen delivery and tissue extraction, not pulmonary gas exchange in isolation (DiBartola 2012). The absence of a statistically significant difference between P0 and P1 in the LG group is consistent with clinical stability. Even with the increase in respiratory rate during the post-anesthetic period, the slight decrease in venous pO_2_ did not result in clinical alterations during the post-anesthetic period.

Similar, or even lower, values have already been reported for the genus *Phrynops*, via intracardiac puncture in *P. hilarii* (Reischl et al. 1984) and, for the species in question, in *P. geoffroanus* via subclavian artery cannulation (Cordeiro 2019), although collection sites differ between studies. Chelonians also exhibit physiological cardiac *shunts* capable of altering blood gases in response to ventilatory changes (Williams and Hicks 2016; Greunz et al. 2018), given that pharmacological reversal involved only dexmedetomidine, at a lower dose than in other studies (Schnellbacher et al. 2012; Cermakova et al. 2017; Masi et al. 2023), together with adaptations already described in the oxygen transport cascade of this group (Porteus et al. 2011). None of these factors alone fully explains the discrete decrease observed, together, however, they support the idea that this alteration found in the present study does not represent a real compromise of respiratory homeostasis.

The limitations of this study include the variability in the timing of anesthesia (day and night), which may have influenced physiological responses. Nociceptive reflexes were not assessed by digital pinching, only by tactile stimulation, which may have limited the assessment of the depth of anesthesia. The animals were not blinded or randomized into blocks, as allocation was performed by simple random selection. Despite these limitations, the study provides fundamental data to support future investigations of anesthetic protocols in *Phrynops geoffroanus*, since the combination of dexmedetomidine and ketamine in the HG group showed higher sedation scores than in the LG group, and the reversal of dexmedetomidine with atipamezole was effective in both groups.

## Data Availability

The data used to generate the results presented in the article are available via email from the corresponding author.
